# The Janus Face of sFRP4 in Cancer: From Mechanistic Complexity to Therapeutic Potential

**DOI:** 10.3390/ijms27135693

**Published:** 2026-06-24

**Authors:** Lingqun Yu, Fei Fang, Minpu Zhang, Ye Li, Mingzhen Li, Changgang Sun, Jing Zhuang, Cun Liu

**Affiliations:** 1 College of Traditional Chinese Medicine, Shandong Second Medical University, Weifang 261000, China; 2 Faculty of Chinese Medicine, Macau University of Science and Technology, Macau 999078, China

**Keywords:** sFRP4, Wnt signaling, cancer, epigenetic regulation, functional duality

## Abstract

Secreted frizzled-related protein 4 (sFRP4) has traditionally been regarded as a Wnt antagonist with tumor-suppressive properties. However, growing evidence indicates that its role in cancer is far more complex and highly context-dependent. Depending on tumor type, molecular subtype, epigenetic state, and microenvironmental conditions, sFRP4 may exert either inhibitory or tumor-promoting effects. This functional heterogeneity has important implications for understanding cancer biology and for evaluating the clinical relevance of sFRP4. In this review, we summarize current knowledge of the structural features, regulatory mechanisms, and signaling functions of sFRP4, and discuss how these factors shape its diverse roles across malignancies. We further examine its potential significance in diagnosis, prognosis, therapeutic stratification, and systemic metabolic regulation. A clearer understanding of the context-specific behavior of sFRP4 may help refine its value as a biomarker and support the development of more precise and mechanism-informed therapeutic strategies.

## 1. Introduction

Secreted frizzled-related proteins (sFRPs) are a family of secreted glycoproteins that have attracted broad attention because of their important roles in development, tissue homeostasis, and disease. Initially characterized mainly as extracellular modulators of Wnt signaling, sFRP family members are now recognized as participants in a wider range of biological processes, including cell proliferation, differentiation, apoptosis, fibrosis, and metabolic regulation. Accordingly, altered expression or dysregulation of sFRPs has been reported in multiple pathological conditions, including cardiovascular disease, metabolic disorders, inflammatory diseases, and cancer. These observations have established the sFRP family as an important group of signaling regulators with substantial pathophysiological and translational relevance.

Among the five known sFRP family members, sFRP4 has drawn particular interest because of its tissue-specific distribution [1], distinctive regulatory characteristics, and broad disease associations [2]. Beyond its roles in endocrine and metabolic regulation, sFRP4 has been increasingly investigated in a variety of human malignancies, where it has been linked to aberrant signaling, epigenetic dysregulation, and heterogeneous clinical outcomes. In this context, the study of sFRP4 also reflects a broader shift in cancer biology. The traditional view that cancer-associated genes function in a strictly oncogenic or tumor-suppressive manner has become increasingly difficult to sustain. In many tumors, gene function is shaped by cellular context, molecular subtype, and microenvironmental conditions, leading to effects that are conditional rather than uniform. This evolving understanding also applies to the sFRP family, which was initially described mainly as a group of extracellular Wnt antagonists with anticancer activity but is now recognized to have more variable and context-dependent functions.

sFRP4 has classically been regarded as a tumor suppressor, largely because of its inhibitory effects on canonical Wnt/β-catenin signaling [3,4]. In many cancers, reduced sFRP4 expression is associated with promoter hypermethylation, Wnt pathway activation, and more aggressive biological behavior, supporting this conventional view [5]. However, accumulating evidence indicates that this model is incomplete. In selected tumor types and molecular contexts, sFRP4 expression may correlate with immune evasion, stromal remodeling, treatment resistance, or unfavorable prognosis [6]. These divergent effects likely reflect the combined influence of tumor-intrinsic signaling states, modular domain-dependent interactions with canonical and non-canonical Wnt pathways, crosstalk with non-Wnt signaling networks, and cues from the tumor microenvironment (TME) [7]. Therefore, sFRP4 should not be viewed simply as a constitutively protective Wnt antagonist, but rather as a context-sensitive regulator whose biological consequences vary across cancer types, disease stages, and microenvironmental settings.

Although sFRP family members and Wnt regulators have been widely discussed, a focused synthesis of the determinants that govern the functional divergence of sFRP4 in cancer remains limited. In this review, we summarize the discovery and structural characteristics of sFRP4, its interactions with canonical and non-canonical Wnt signaling, and the epigenetic and microenvironmental mechanisms that shape its context-specific activity. We further examine how these regulatory layers influence the heterogeneous expression patterns and biological consequences of sFRP4 across tumor types, and assess its potential as a biomarker for diagnosis, prognosis, therapeutic stratification, and systemic metabolic regulation. Particular attention is given to the translational implications of its dual behavior, including the need for context-aware therapeutic modulation and the opportunities, as well as the limitations, of emerging strategies such as recombinant proteins and nanocarrier-based delivery platforms.

## 2. Discovery and Structural Features of sFRP4

**Figure 1:**
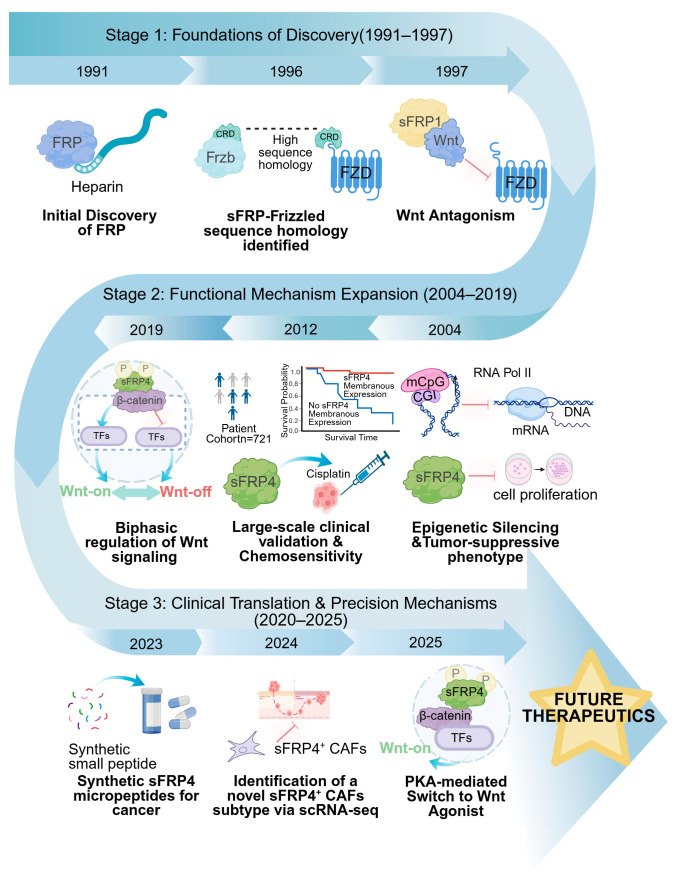
Historical milestones of sFRP4: from molecular discovery to therapeutic targeting. Created in BioRender. LQ, Y. (2026) https://BioRender.com/kybvnkk (accessed on 5 February 2026).

Literature retrieval for this study was systematically performed across major electronic databases, including PubMed and Web of Science, utilizing “sFRP4” and “secreted frizzled-related protein 4” as the core search terms, with relevant evidence from the broader sFRP family also supplemented where appropriate. The retrieved results were comprehensively summarized and analyzed to ensure structural clarity and objectivity throughout this literature synthesis. Research has shown that the human sFRP family consists of five secreted glycoproteins, designated sFRP1–5, which are broadly divided into two subfamilies on the basis of sequence homology: sFRP1/2/5 and sFRP3/4. Interest in this protein family began in 1991, when an sFRP-related gene was first identified in human embryonic lung fibroblast cell lines [8,9], providing an early basis for subsequent studies of secreted Wnt modulators (Figure 1). Additional mammalian homologues were later identified through expressed sequence tag database screening for Frizzled (FZD)-related sequences [7,10,11], and sFRP4 itself was subsequently cloned in 1997 [11].

The sFRP4 gene is located on the short arm of chromosome 7, spans approximately 11 kb, and contains six exons encoding a 346-amino acid protein with a molecular weight of about 39.9 kDa [12]. Like other sFRP family members, sFRP4 contains two principal domains: an N-terminal cysteine-rich domain (CRD) and a C-terminal netrin-related motif [10]. The CRD shares marked homology with the Wnt-binding region of Frizzled receptors, a feature that originally placed sFRP4 within the framework of extracellular Wnt antagonism [13]. The C-terminal region, however, has also been implicated in broader protein interactions and may contribute to functions that are not captured by a ligand-sequestration model alone.

This domain organization provides an initial basis for understanding why sFRP4 does not behave as a uniform signaling inhibitor in all settings. Although it has traditionally been described as an antagonist that limits Wnt pathway activation by competing for Wnt ligands or receptor engagement, this explanation appears incomplete in the context of cancer. Experimental observations from different systems suggest that the biological effects of sFRP family proteins can vary with molecular context, local signaling conditions, and protein abundance, raising the possibility that sFRP4 may influence Wnt signaling in more than one way. These structural and functional considerations make sFRP4 a plausible mediator of context-dependent signaling output and set the stage for the mechanistic complexity discussed in later sections.

## 3. sFRP4 as a Key Modulator of Wnt Signaling

Owing to its structural homology to Frizzled-related domains, sFRP4 is generally regarded as an important extracellular regulator of the Wnt signaling network, a highly conserved pathway involved in embryonic development, tissue homeostasis, and cell fate control [7]. In the conventional model, sFRP4 functions mainly as a negative regulator by interacting with Wnt ligands and Frizzled (FZD) receptors, thereby limiting Wnt signal transmission [14]. This antagonistic view is supported in part by observations that sFRP4 can reduce β-catenin accumulation and attenuate the expression of Wnt-responsive genes associated with tumor progression [15].

**Figure 2:**
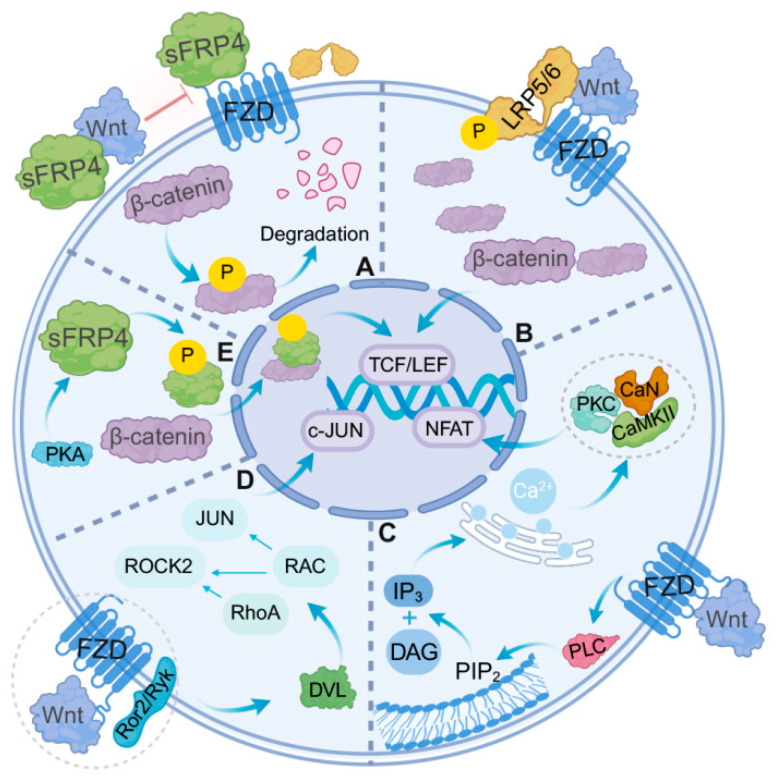
Wnt Signaling Pathways and sFRP4-Mediated Modulation. (**A**) In normal cells, sFRP4 binds to Wnt proteins, preventing their interaction with downstream FZD and LRP5/6 receptors. When the canonical Wnt/β-catenin signaling pathway is inactivated, cytoplasmic β-catenin is recognized and phosphorylated, and the phosphorylated β-catenin is subsequently degraded. (**B**) Wnt ligands bind to the FZD receptors and LRP5/6 co-receptors on the cell membrane, inhibiting the β-catenin destruction complex. This leads to the accumulation of β-catenin in the cytoplasm and its translocation into the nucleus, where β-catenin associates with TCF/LEF transcription factors to initiate the transcription of downstream target genes. (**C**) Following the binding of Wnt proteins to FZD receptors, PLC is activated via G proteins. PLC hydrolyzes PIP_2_ on the cell membrane to generate IP_3_ and DAG. IP_3_ triggers the release of Ca^2+^ from the endoplasmic reticulum, and intracellular Ca^2+^ further activates kinases including PKC, CaMKII and CaN, which in turn activate the NFAT transcription factor and promote its nuclear translocation. (**D**) Wnt proteins bind to FZD receptors and cooperate with Ror2/Ryk receptors to recruit and activate DVL, which subsequently activates the small GTPases RhoA and RAC. RhoA stimulates ROCK2 kinase activity, while RAC triggers the JNK pathway, both of which drive the nuclear translocation of TFs such as c-JUN. (**E**) sFRP4 can be phosphorylated by PKA. The phosphorylated sFRP4 binds to β-catenin and translocates into the nucleus, thereby enhancing the transcriptional activity of LEF/TCF complexes. Created in BioRender. LQ, Y. (2026) https://BioRender.com/xhpi0d3 (accessed on 5 February 2026).

At the same time, the relationship between sFRP4 and Wnt signaling cannot be fully understood within a uniformly inhibitory framework. Findings from different experimental and tumor contexts suggest that its effects may vary according to cellular background, pathway status, and microenvironmental conditions. Rather than acting as a fixed antagonist, sFRP4 appears to influence Wnt signaling in a context-dependent manner, with consequences that may differ between canonical and non-canonical branches and may extend to signaling pathways beyond Wnt itself. The following sections therefore examine these regulatory modes separately to clarify how sFRP4 shapes signaling output in cancer (Figure 2).

### 3.1. sFRP4 and Canonical Wnt/β-Catenin Signaling

sFRP4 is commonly described as an extracellular antagonist of canonical Wnt/β-catenin signaling. In many cancers, its expression is reduced or silenced through promoter hypermethylation, which is widely regarded as a major mechanism of transcriptional repression. Under conditions in which sFRP4 is preserved or restored, it has generally been associated with inhibition of Wnt/β-catenin signaling through interference with ligand–receptor interactions and restriction of downstream β-catenin stabilization. In hepatocellular carcinoma models, sFRP4 suppressed malignant progression in parallel with increased GSK-3β expression and reduced β-catenin expression [16]. These findings support the view that sFRP4 can act, at least in some settings, as a negative regulator of canonical Wnt signaling.

At the same time, the relationship between sFRP4 and canonical Wnt signaling is unlikely to be uniformly inhibitory in all biological contexts. The available evidence suggests that the effects of sFRP proteins may depend on factors such as ligand availability, local protein concentration, receptor composition, and microenvironmental conditions. In this regard, observations from other sFRP family members are informative, even though they should not be taken as direct evidence for sFRP4 itself. In Xenopus embryos, Frzb and Crescent were shown to bind extracellular Wnt8 and Wnt11, respectively, facilitating Wnt diffusion and enhancing signaling output [17]. Likewise, sFRP2 has been reported to form complexes with Wnt, increase its accumulation on cell-surface heparan sulfate proteoglycans, and promote endocytosis together with exosome-mediated Wnt re-secretion [18]. These studies indicate that sFRP-mediated regulation of canonical Wnt signaling may involve redistribution, presentation, or stabilization of Wnt ligands rather than simple neutralization alone.

Interactions among sFRP family members may add a further layer of complexity to pathway regulation [19]. During rat renal organogenesis, sFRP1 and sFRP2 displayed both competitive and cooperative effects, and sFRP2 was associated with enhanced Wnt signaling [20]. sFRPs may also engage Frizzled receptors in ways that favor downstream signal propagation under specific conditions. Consistent with this possibility, sFRP2 has been reported to enhance Wnt3a-induced transcriptional activity [21]. In a mouse model, sFRP2 expression was directly regulated by TRα1 and promoted β-catenin signaling through Frizzled-dependent mechanisms, resulting in β-catenin stabilization, induction of downstream target genes, and increased cell proliferation [22]. A concentration-dependent biphasic effect has also been described for sFRP1 in Armadillo stabilization assays, in which low concentrations enhanced Wnt signaling whereas high concentrations inhibited it [23]. Distinct ligand preferences may further contribute to these divergent effects; for example, Frzb inhibits Wnt1 and Wnt8 but not Wnt3a [24,25].

For sFRP4 specifically, these family-level observations are best viewed as a conceptual framework rather than as direct mechanistic proof. They help explain why a purely antagonistic model may be insufficient, but they do not establish that sFRP4 itself consistently enhances canonical Wnt signaling in cancer. At present, the strongest evidence still supports an inhibitory role for sFRP4 in canonical Wnt and β-catenin signaling, particularly through limiting β-catenin accumulation and attenuating the expression of Wnt-responsive genes associated with tumor progression, whereas any Wnt-activating effect of sFRP4 appears likely to be context-restricted and remains to be more clearly defined in specific tumor models, cellular compartments, and microenvironmental settings.

### 3.2. sFRP4 in Non-Canonical Wnt and Related Signaling Contexts

In addition to its effects on canonical Wnt/β-catenin signaling, sFRP4 has been implicated in non-canonical Wnt signaling and in a broader set of tumor-associated signaling responses. Evidence from several experimental systems suggests that its biological activity cannot be accounted for solely by β-catenin-dependent mechanisms. In β-catenin-deficient mesothelioma cells, for example, sFRP4-induced apoptosis has been linked to β-catenin-independent signaling, including pathways associated with c-Jun N-terminal kinase (JNK) [26]. In glioblastoma models, sFRP4 has likewise been reported to promote tumor cell apoptosis in association with Fas–p53 activation, Wnt/Ca^2+^ signaling, and reactive oxygen species (ROS)-related responses [27,28]. Together, these findings support the view that sFRP4 can engage signaling outputs beyond the canonical β-catenin axis.

Additional support for this interpretation comes from domain-based functional studies. Both the cysteine-rich domain (CRD) and the netrin-related region of sFRP4 have been reported to increase intracellular calcium levels and activate Wnt/Ca^2+^ signaling, with subsequent suppression of angiogenic factor transcription. These observations broaden the functional scope of sFRP4 beyond tumor cell proliferation and survival, and suggest that it may also influence angiogenesis-related programs. Beyond these domain-mediated effects, sFRP4 has also been shown to modulate Wnt signaling through a phosphorylation-dependent mechanism. Specifically, protein kinase A (PKA) can phosphorylate sFRP4, and the phosphorylated form of sFRP4 binds to β-catenin and translocates into the nucleus, where it enhances the transcriptional activity of LEF/TCF complexes and promotes the expression of stemness-related genes [2,29].

The available evidence further indicates that sFRP4 may intersect with signaling pathways involved in apoptosis, oxidative stress responses, and vascular remodeling. In glioblastoma models, the pro-apoptotic effects associated with sFRP4 were observed in a signaling environment that included ROS accumulation, death receptor activation, and calcium-dependent responses [27,28]. Its anti-angiogenic activity also raises the possibility that sFRP4 affects communication between tumor cells and stromal or endothelial compartments, thereby shaping the local signaling environment rather than acting only through an isolated intracellular pathway. Although the current data remain limited, they support a broader view of sFRP4 as a component of interconnected signaling networks rather than a regulator confined to a single Wnt branch.

This wider signaling perspective is important for understanding why the downstream consequences of sFRP4 expression may differ across tumors even when upstream Wnt-related inputs appear similar. The current literature does not yet define these non-canonical and Wnt-adjacent effects with the same clarity as its canonical inhibitory role, but it does indicate that the biological actions of sFRP4 extend beyond β-catenin-centered signaling alone.

## 4. Epigenetic Regulation of sFRP4 in Cancer

Epigenetic regulation is a major determinant of gene expression heterogeneity in cancer and appears to be particularly relevant to sFRP4. DNA methylation, histone modifications, and non-coding RNAs together form a multilayered regulatory system that can influence sFRP4 expression across different tumor types and disease stages. In many malignancies, reduced sFRP4 expression has been linked to promoter hypermethylation, repressive chromatin states, and post-transcriptional suppression, findings that are generally consistent with loss of its growth-restraining activity. At the same time, the epigenetic control of sFRP4 is not uniform. In some settings, distinct regulatory states are associated with preserved or even increased expression. In gastric cancer, for example, promoter demethylation has been linked to sFRP4 upregulation, which may contribute to context-dependent tumor-promoting effects through altered interactions within the Wnt signaling network [6].

The regulation of sFRP4 also extends beyond transcription alone. In some tumor contexts, mRNA abundance does not parallel protein expression, pointing to an additional contribution from post-transcriptional or translational mechanisms. In the prostate tumor microenvironment, stromal sFRP4 mRNA expression has been reported to increase, whereas sFRP4 protein levels are reduced, a pattern associated with a local environment permissive for tumor invasion [30]. This discrepancy suggests that miRNA-mediated repression, altered translation, or changes in protein stability may further shape the biological output of sFRP4. These observations indicate that the expression and function of sFRP4 are regulated at multiple levels, providing an important framework for the following sections on DNA methylation, chromatin regulation, and post-transcriptional control.

### 4.1. DNA Methylation

**Figure 3:**
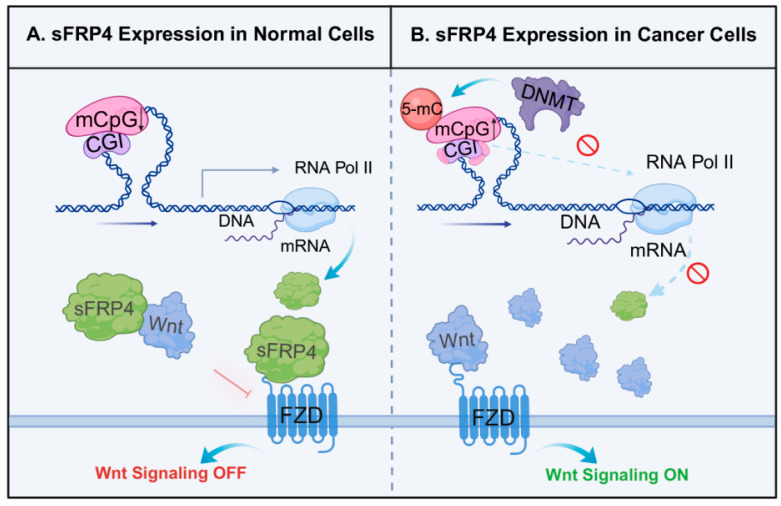
DNA methylation regulates sFRP4. (**A**) In normal cells, the promoter region of the sFRP4 gene is hypomethylated, enabling efficient transcription and translation into the sFRP4 protein, which exerts a tumor-suppressive effect by inhibiting Wnt signaling. (**B**) In cancer cells, aberrant activation of DNMT frequently induces hypermethylation of CpG islands within the sFRP4 promoter. This hypermethylation impedes transcription factor binding to the promoter, repressing sFRP4 transcription and resulting in gene silencing. Created in BioRender. LQ, Y. (2026) https://BioRender.com/79ylaib (accessed on 5 February 2026).

DNA methylation is one of the best-characterized epigenetic mechanisms involved in transcriptional repression in cancer [31,32,33]. For sFRP4, altered promoter methylation has emerged as a recurrent explanation for reduced expression across multiple tumor types. In non-malignant settings, CpG-rich regions within the sFRP4 promoter are generally compatible with active transcription, whereas in tumor cells, increased DNA methyltransferase (DNMT) activity is frequently associated with promoter hypermethylation [26,34,35] (Figure 3). This shift can interfere with transcription factor access and contribute to reduced sFRP4 expression [36]. In many contexts, such repression is consistent with diminished restraint on Wnt-related signaling and may favor tumor progression. The extent and distribution of methylation are also likely to influence transcriptional output, with more extensive CpG island hypermethylation being more often associated with stable silencing than more localized methylation changes.

Several experimental observations are consistent with this regulatory model. In cervical cancer cell lines, silencing of DNMT1 reduced methylation of the sFRP4 promoter and was accompanied by increased sFRP4 mRNA expression [34]. Promoter hypermethylation has likewise been reported as a frequent mechanism of sFRP4 silencing in multiple tumor types [37]. Related findings in endocrine-associated malignancies, including breast, prostate, and ovarian cancers, further support the broader relevance of promoter methylation for the downregulation of sFRP family genes. In hypermethylated cancer cell models, treatment with demethylating agents restored sFRP expression, including sFRP4, suggesting that this regulatory axis is at least partly reversible [38].

Clinical data also support the pathological relevance of sFRP4 methylation. In cervical cancer, colorectal cancer, and esophageal adenocarcinoma, sFRP4 methylation status has been associated with aggressive clinicopathological features or less favorable outcomes [39]. Although the consistency and strength of these associations vary among tumor types, the available evidence identifies promoter methylation as a major contributor to dysregulated sFRP4 expression in cancer and as a plausible link between epigenetic silencing and tumor phenotype.

### 4.2. Histone Modification

Beyond DNA methylation, histone-associated regulation may provide an additional mechanism contributing to dysregulated sFRP4 expression in cancer [40,41]. Compared with promoter methylation, however, direct evidence linking histone modifications to sFRP4 remains more limited. Even so, the available data suggest that chromatin-level repression may help explain sFRP4 downregulation in settings where promoter methylation alone does not fully account for its expression status.

**Figure 4:**
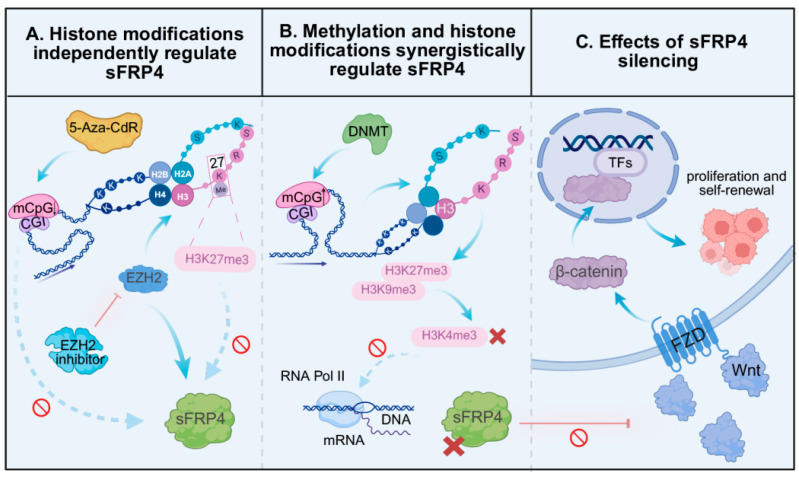
Histone modifications regulate sFRP4. (**A**) sFRP4 silencing is driven by EZH2-mediated H3K27me3, rather than DNA methylation, as demethylating treatment with 5-Aza-CdR does not restore its expression. Administration of an EZH2 inhibitor alone is sufficient to reactivate sFRP4 expression. (**B**) The CpG island in the sFRP4 promoter region undergoes hypermethylation catalyzed by DNMT. Under the action of enzyme complexes recruited by DNA hypermethylation, histone modifications occur in the sFRP4 promoter region, leading to extensive chromatin condensation into heterochromatin. This prevents the binding of TFs and RNA polymerase II, resulting in transcriptional silencing of the gene. (**C**) Following sFRP4 silencing, Wnt ligands can bind to FZD receptors unimpeded, activating downstream signaling to drive cytoplasmic accumulation and nuclear translocation of β-catenin. Nuclear β-catenin then interacts with TFs, initiating the expression of target genes and ultimately promoting aberrant cell proliferation and self-renewal. Created in BioRender. LQ, Y. (2026) https://BioRender.com/79ylaib (accessed on 5 February 2026).

A notable example comes from colorectal cancer, in which the histone methyltransferase EZH2 was reported to regulate sFRP4 expression without altering the DNA methylation status of the sFRP4 promoter [42]. This finding is important because it indicates that repression of sFRP4 may occur through histone-mediated mechanisms independently of promoter hypermethylation in at least some tumor contexts. More broadly, it suggests that loss of sFRP4 expression cannot always be inferred from DNA methylation status alone and that distinct epigenetic routes may converge on the same transcriptional outcome (Figure 4).

Histone-associated repression may also cooperate with promoter methylation to reinforce sFRP4 silencing. In endocrine-related malignancies, including prostate and breast cancer, combined epigenetic repression involving DNA methylation and histone-associated mechanisms has been linked to downregulation of sFRP family members, with evidence that sFRP4 may participate in this broader pattern [38]. These observations support a model in which histone modifications do not necessarily act in isolation but may stabilize or deepen transcriptional suppression initiated through other epigenetic changes. At present, the specific histone marks involved, their upstream regulators, and their relative contribution across tumor types are not yet defined with the same clarity as promoter methylation.

### 4.3. Non-Coding RNA

In addition to promoter methylation and histone-associated regulation, post-transcriptional control provides another layer through which sFRP4 expression may be modulated in cancer. Among non-coding RNAs, the clearest evidence currently concerns microRNAs (miRNAs), several of which have been implicated in tumor progression in association with reduced sFRP4 expression and increased Wnt-related signaling activity.

In pancreatic ductal adenocarcinoma (PDAC), miR-135b-5p has been reported to show an inverse correlation with sFRP4 expression. Increased miR-135b-5p levels in tumor tissues were associated with reduced sFRP4 expression, alongside increased β-catenin activity and enhanced proliferative and migratory phenotypes. Mechanistically, miR-135b-5p was shown to target the 3′ untranslated region (3′-UTR) of sFRP4, supporting a direct post-transcriptional mode of repression [43].

**Figure 5:**
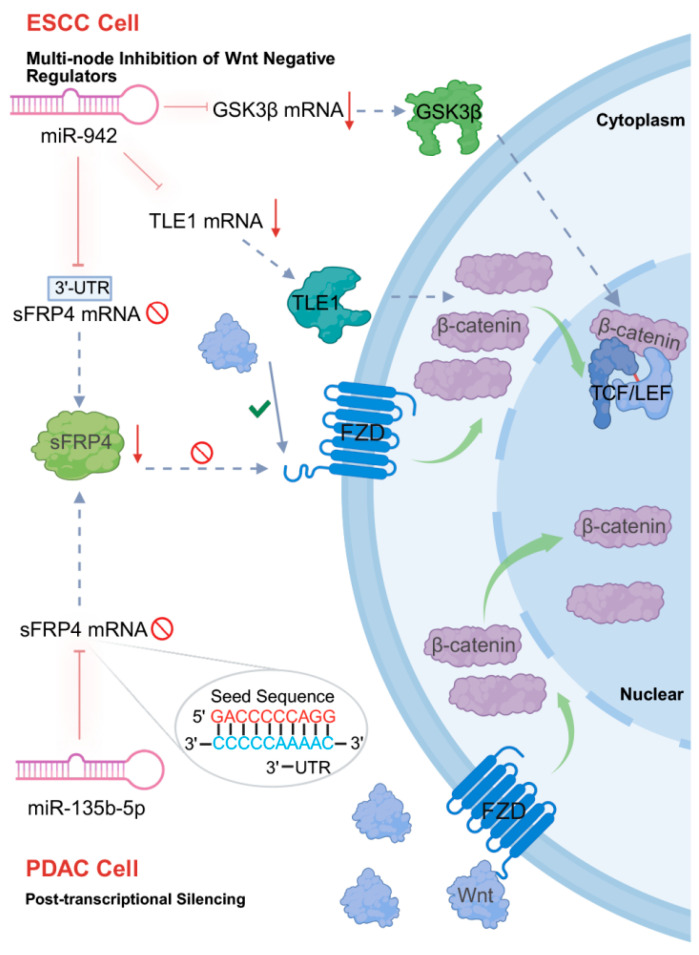
Non-coding RNA regulates sFRP4. In ESCC, sFRP4 inhibits the initiation of the Wnt pathway by binding to Wnt proteins, thereby preventing their interaction with the membrane receptors Frizzled/LRP5/6. miR-942 directly targets the 3′-UTR of sFRP4, reducing its mRNA stability and protein expression levels, without antagonizing the Wnt pathway, which remains normally activated. In PDAC, aberrant upregulation of miR-135b-5p directly suppresses sFRP4 expression. This silencing subsequently induces the pathological upregulation of β-catenin. Created in BioRender. LQ, Y. (2026) https://BioRender.com/y02p2xi (accessed on 5 February 2026).

A related but broader pattern has been described in esophageal squamous cell carcinoma (ESCC), where miR-942 promotes tumor progression through coordinated regulation of multiple Wnt pathway components. In this setting, miR-942 has been reported to suppress sFRP4 while also downregulating other negative regulators of Wnt signaling, including GSK3β and TLE1 [44] (Figure 5). This finding is notable because it suggests that miRNA-mediated enhancement of Wnt output may arise not only from repression of sFRP4 itself, but also from simultaneous targeting of several inhibitory nodes within the same signaling network.

Current evidence therefore supports miRNA-mediated repression as a relevant component of the broader regulatory landscape governing sFRP4 expression. At the same time, this layer of regulation remains less comprehensively defined than promoter methylation, and the available data are still centered on a limited number of miRNAs. Whether other classes of non-coding RNAs, including long non-coding RNAs or circular RNAs, also contribute to sFRP4 regulation in cancer remains largely unresolved.

## 5. Context-Dependent Roles of sFRP4 Across Tumor Types

sFRP4 is widely regarded as a tumor-suppressive factor in many cancer settings, but this view does not fully capture the diversity of its biological effects across tumor types. In several experimental models, exogenous sFRP4 has been shown to activate caspase-3/7 and promote apoptosis, including in breast cancer cells [45], supporting a growth-restraining role under certain conditions. At the same time, accumulating evidence indicates that the consequences of sFRP4 expression are shaped by tumor lineage, cellular differentiation state, tissue compartment, and disease stage.

This complexity is reflected in observations that do not fit a uniformly tumor-suppressive model. In esophageal cancer, sFRP4 has been linked to embryonic-like programs and stem/progenitor-associated phenotypes, suggesting that its expression may in some contexts accompany cellular states associated with tumor plasticity rather than simple growth inhibition [46]. Functional divergence may also arise between tumor and stromal compartments. In breast lobular tumors, stromal expression of Wnt5a and sFRP4 increases with tumor grade [47], implying that sFRP4 may participate in a microenvironment-dependent signaling context associated with disease progression.

**Figure 6:**
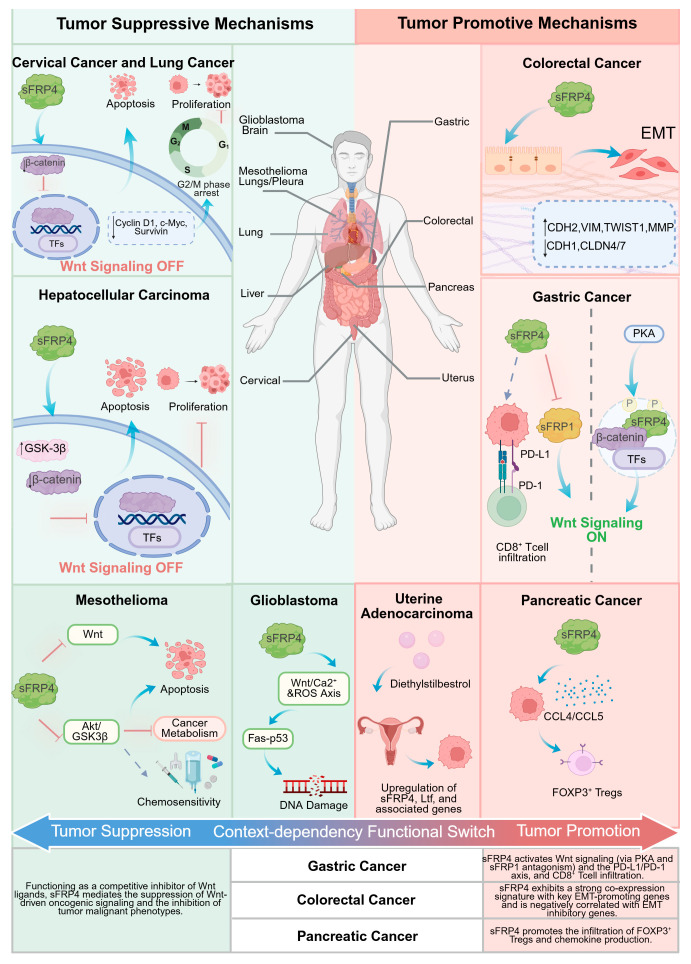
Dual functional roles of sFRP4 as a context-dependent molecular hub in malignancy. This figure provides a systematic overview of the molecular mechanisms underlying the dual tumor-suppressive and oncogenic roles of sFRP4, based on representative cancer models with unique regulatory characteristics. By integrating tissue-specific signals across diverse cancer models, sFRP4 exerts dual functional roles characterized by either multi-axial tumor suppression or paradoxical oncogenic promotion. In tumor-suppressive contexts, sFRP4 inhibits malignant phenotypes by antagonizing canonical Wnt signaling (cervical cancer, lung cancer, and hepatocellular carcinoma), activating the Fas-p53 axis (glioblastoma), and inhibiting the Akt/GSK-3β pathway (mesothelioma). Conversely, in oncogenic settings, sFRP4 manifests multidimensional pro-tumor activities: activating Wnt signaling via PKA phosphorylation and sFRP1 antagonism (gastric cancer), driving the EMT program (colorectal cancer), and inducing an immunosuppressive microenvironment (pancreatic cancer). Furthermore, in diethylstilbestrol-induced models, sFRP4 upregulation acts as a potential pathological driver for uterine adenocarcinoma initiation. These divergent mechanisms underscore the profound functional plasticity of sFRP4, providing a foundation for further exploring the molecular structure and microenvironmental determinants governing its functional switch. Created in BioRender. LQ, Y. (2026) https://BioRender.com/kybvnkk (accessed on 5 February 2026).

These findings frame sFRP4 as a context-sensitive regulator rather than a uniformly acting tumor suppressor. Across malignancies, its biological effects appear to depend not only on cancer type, but also on the cellular compartment in which it is expressed and the stage-specific signaling environment in which it operates (Figure 6). This perspective provides the basis for understanding how sFRP4 can be associated with either tumor-restraining or tumor-supportive phenotypes in different oncological settings.

To avoid treating heterogeneous findings as equivalent evidence and to provide an operational synthesis, Table 1 summarizes the functional roles of sFRP4 across different cancer types and contexts, with explicit columns for assay modalities, evidence types, and limitations. Overall, sFRP4 is more consistently interpreted as a tumor-restraining factor when epigenetically silenced in tumor cells and where its restoration suppresses Wnt-related malignant phenotypes. Conversely, tumor-supportive interpretations are more plausible when sFRP4 is enriched in mesenchymal, immune-remodeled, or advanced stromal contexts associated with extracellular matrix remodeling, treatment resistance, or poor survival. Importantly, because promoter methylation, tissue transcripts, protein abundance, and clinical survival associations represent distinct, non-interchangeable evidence types, contradictory findings across malignancies are best reconciled by systematically considering assay modality, cellular compartment, and clinical endpoint.

### 5.1. Solid Tumors

#### 5.1.1. Gynecological Tumors

Among gynecological malignancies, the tumor-suppressive role of sFRP4 is most strongly supported in cervical cancer, where its downregulation is closely linked to epigenetic silencing. Aberrant promoter hypermethylation appears to be a major mechanism underlying reduced sFRP4 expression. In cervical adenocarcinoma, promoter hypermethylation of sFRP4 was detected in 65.2% (15/23) of tumor tissues but was absent in normal cervical swab samples [48]. Bisulfite sequencing further supported sFRP4 promoter hypermethylation as a characteristic epigenetic alteration in this tumor type. Notably, methylation status was not significantly associated with clinical stage, a pattern consistent with the possibility that this alteration occurs relatively early during disease development [48].

A similarly progressive pattern has been reported in cervical squamous lesions and cervical squamous cell carcinoma (SCC). The methylation frequencies of the sFRP4 promoter in normal tissues, low-grade squamous intraepithelial lesions, high-grade squamous intraepithelial lesions, and SCC were 0%, 4.4%, 36.7%, and 67.9%, respectively [49]. This stepwise increase supports the view that sFRP4 methylation accompanies malignant progression in cervical SCC and may have utility as a molecular biomarker in cervical tumor screening.

Mechanistic studies further strengthen this interpretation. In cervical cancer cell models, DNMT1 knockdown or treatment with 5-aza-2′-deoxycytidine reduced sFRP4 promoter methylation and restored sFRP4 mRNA expression [34]. These findings indicate that DNMT1 contributes to sFRP4 silencing through maintenance of promoter methylation and suggest that this repression may be at least partly reversible. In this context, the early and progressive nature of sFRP4 methylation also provides a rationale for its potential translational relevance. Bioinformatic analyses have suggested that sFRP4, together with APOD and ACKR1, may be associated with survival outcomes in cervical cancer, supporting possible prognostic value. sFRP4 has also been proposed as part of a multi-gene panel including other sFRP family members to improve molecular detection strategies, particularly in cervical adenocarcinoma [48].

Outside cervical cancer, the available evidence still points predominantly toward a tumor-restraining role for sFRP4 across several gynecological tumors, although the supporting data are less extensive. In uterine leiomyosarcoma, sFRP4 reduced cell survival and migratory capacity while enhancing cell adhesion, consistent with inhibition of invasive behavior [57]. In endometrial cancer, the PBX1/sFRP4 axis has been implicated in suppression of invasion and metastasis. PBX1 directly binds the sFRP4 promoter and enhances its transcription, thereby inhibiting Wnt/β-catenin signaling, reducing metastasis-related protein expression, and increasing E-cadherin levels, with an overall suppressive effect on epithelial–mesenchymal transition [50]. Rescue experiments further support sFRP4 as a key downstream mediator of PBX1-dependent tumor suppression [50]. In serous ovarian cancer, recombinant sFRP4 has likewise been reported to inhibit aberrant Wnt signaling and suppress tumor cell proliferation and invasion [53].

At the same time, this pattern is not entirely uniform across all gynecological contexts. In a diethylstilbestrol-induced mouse model of uterine adenocarcinoma, increased sFRP4 expression was observed together with lactoferrin upregulation during tumor development [56]. This association raises the possibility that sFRP4 may participate differently in specific hormonal or developmental contexts, although it does not by itself establish a tumor-promoting function and its precise contribution remains to be defined.

#### 5.1.2. Digestive System Tumors

Digestive system tumors provide some of the clearest illustrations of the context-dependent roles of sFRP4. Across this group of malignancies, sFRP4 may be epigenetically silenced and behave in a tumor-restraining manner in some settings, yet remain expressed or become re-elevated in others, where it may instead associate with stromal signaling, immune remodeling, or more aggressive disease states.

An early methylation-driven silencing pattern is evident in esophageal adenocarcinoma (EAC) and its precursor lesion, Barrett’s esophagus. sFRP4 promoter methylation was detected in 73% of EAC tissues and 78% of Barrett’s epithelial tissues, compared with a much lower frequency in normal esophageal tissues [39]. These findings suggest that sFRP4 methylation is a common early event in the Barrett’s esophagus-EAC sequence and may contribute to malignant transformation.

A more classically tumor-suppressive profile has been described in hepatocellular carcinoma (HCC). In this setting, sFRP4 overexpression reduced cell viability and proliferation, accompanied by upregulation of GSK-3β and downregulation of β-catenin, findings consistent with inhibition of Wnt/β-catenin signaling [16].

Pancreatic ductal adenocarcinoma (PDAC), by contrast, highlights compartment-dependent divergence. At the epithelial-cell level, promoter hypermethylation of sFRP4 has been detected in PDAC tissues and associated with reduced expression and greater tumor invasiveness [72]. In addition, miR-135b-5p directly targets the 3′ untranslated region (3′-UTR) of sFRP4 mRNA, suppressing its expression, enhancing Wnt/β-catenin signaling, and promoting malignant phenotypes [43]. These findings support a tumor-suppressive interpretation of sFRP4 within the malignant epithelial compartment. However, analyses of clinical samples, public datasets, and KPC mouse models have shown increased sFRP4 expression during progression from pancreatic intraepithelial neoplasia to PDAC, particularly in association with stromal and microenvironmental changes [73]. Elevated sFRP4 has been linked to chemokine secretion, FOXP3^+^ regulatory T-cell infiltration, and an immunosuppressive tumor microenvironment [73]. Activated pancreatic stellate cells have also been reported to secrete sFRP4 in a paracrine manner, whereas retinoic acid reduces sFRP4 release from these cells and attenuates Wnt/β-catenin signaling [74]. Additionally, adipose stromal cells (ASCs) can serve as a source of sFRP4 in the tumor microenvironment. Recent evidence has demonstrated that upon co-culture with pancreatic cancer cells, ASCs undergo a fibroblastic transition driven by Wnt and TGFβ signaling, during which SFRP4 expression is markedly induced. Functional ablation of SFRP4 in ASCs suppressed cancer cell migration and invasion in vitro, while in vivo tumors in SFRP4-knockout mice exhibited reduced desmoplasia, less epithelial dedifferentiation, decreased growth rate, and diminished metastatic progression. These findings further support a tumor-promoting role of stroma-derived sFRP4 in pancreatic cancer and suggest SFRP4 as a potential therapeutic target [75]. Together, these observations suggest that the biological significance of sFRP4 in PDAC differs substantially between tumor cells and stromal compartments.

Colorectal cancer (CRC) similarly illustrates stage- and subtype-dependent behavior. Promoter hypermethylation and reduced expression of sFRP4 are commonly observed in CRC and are particularly evident during disease progression in some cell line models, supporting a role for sFRP4 loss in colorectal tumorigenesis [69]. In mismatch repair-deficient CRC, higher sFRP4 expression has been reported to correlate negatively with Ki-67, again consistent with a tumor-restraining association [70]. However, this pattern is not uniform across all CRC subtypes. In mesenchymal CRC, sFRP4 shows strong co-expression with EMT-promoting genes and an inverse relationship with EMT-inhibitory genes. In this specific subtype, high sFRP4 expression was associated with worse overall survival, suggesting that its significance may reverse in transcriptional contexts characterized by mesenchymal transition and invasive behavior [71].

Gastric cancer presents a similarly bidirectional but even more multilayered pattern. In many gastric tumors, sFRP4 promoter hypermethylation co-occurs with epigenetic inactivation of APC and other sFRP family members, supporting a model in which sFRP4 silencing contributes to Wnt pathway activation and tumor progression [60,61,62,63,64]. In more advanced or context-specific settings, however, increased sFRP4 expression has been linked to tumor-promoting effects. Hypomethylation-associated upregulation of sFRP4 has been proposed to enhance Wnt signaling, in part through antagonism of sFRP1-mediated restraint [6]. In addition, PKA-mediated phosphorylation of sFRP4 has been reported to promote Wnt/β-catenin activation and stem-like features in gastric cancer [29], consistent with earlier observations that phosphorylated sFRP4 can interact with β-catenin- and TCF4-related transcriptional machinery [65]. Beyond direct signaling effects, sFRP4 expression in gastric cancer has also been associated with increased PD-L1 expression and an immune context suggestive of T-cell dysfunction, pointing to a possible link between sFRP4 and immune evasion [66].

Taken as a whole, digestive system tumors make it particularly clear that no single model adequately explains sFRP4 function. Within this organ group, its role appears to vary according to epigenetic state, tumor subtype, disease stage, and the relative contribution of tumor cell-intrinsic versus microenvironment-associated signaling.

#### 5.1.3. Head and Neck Tumor

Current evidence in head and neck malignancies, although limited, mainly supports an epigenetic silencing pattern for sFRP4, particularly in oral squamous cell carcinoma (OSCC). In one study of paraffin-embedded oral cancer samples, sFRP4 promoter methylation was significantly associated with OSCC, with a methylation frequency of 78.38% compared with 33.33% in normal control tissues [77]. This finding is consistent with loss of sFRP4 expression as a frequent event in OSCC.

The potential significance of this alteration is strengthened by the broader molecular context of OSCC. Although mutations in canonical Wnt pathway genes such as APC and Axin are relatively uncommon in this tumor type, aberrant cytoplasmic and nuclear accumulation of β-catenin is frequently observed and has been linked to poor differentiation, enhanced invasiveness, and unfavorable prognosis. In this setting, promoter methylation-mediated loss of sFRP4 provides a plausible alternative mechanism for sustained Wnt pathway activation and β-catenin dysregulation [77]. Rather than arising primarily from classic intracellular pathway mutations, Wnt activation in OSCC may therefore depend in part on epigenetic derepression caused by loss of extracellular antagonists such as sFRP4.

Taken together, the available data support a predominantly tumor-restraining interpretation of sFRP4 in OSCC, while also indicating that epigenetic inactivation may be a particularly relevant route to pathway dysregulation in this disease.

#### 5.1.4. Thoracic Tumor

Current evidence in thoracic malignancies largely supports a tumor-restraining role for sFRP4, although the mechanisms involved vary across tumor types and cellular contexts. This pattern is most extensively documented in malignant mesothelioma (MM), where epigenetic silencing of sFRP family members, including sFRP4, appears to be a frequent event.

Compared with normal pleural tissues, expression of multiple sFRP family genes was downregulated in 85% (18/21) of primary MM samples. Among these, promoter methylation of sFRP4, sFRP1, and sFRP5 was detected at frequencies exceeding 80%, indicating that epigenetic inactivation of extracellular Wnt antagonists is a common alteration in MM [79]. Functional re-expression studies further showed that restoration of sFRP constructs in MM cell lines lacking endogenous sFRP expression induced apoptosis and inhibited cell growth, supporting a tumor-suppressive role for this family in mesothelioma biology [79]. Within this broader context, sFRP4 emerges as a relevant contributor to Wnt pathway restraint rather than as an isolated marker.

More sFRP4-specific studies reinforce this interpretation while also expanding its mechanistic scope. In β-catenin-deficient mesothelioma, sFRP4 was silenced through promoter hypermethylation, and its re-expression was shown to inhibit Wnt signaling through a β-catenin-independent non-canonical pathway [26]. This finding is particularly informative because it suggests that sFRP4-mediated tumor suppression may remain relevant even in tumors lacking detectable β-catenin expression. In mesothelioma JU77 cells, sFRP4 also exerted anticancer effects through modulation of Akt/GSK3β phosphorylation downstream of Wnt signaling, markedly inhibiting cell proliferation and counteracting the pro-proliferative effects of Wnt3a [80]. Notably, this effect did not depend on major metabolic reprogramming such as altered ATP production or glucose utilization. Instead, sFRP4 increased chemosensitivity in part through downregulation of COXIV, suggesting a potential therapeutic relevance in chemoresistant mesothelioma [80].

Breast cancer likewise supports a predominantly tumor-suppressive interpretation of sFRP4, but in a more compartment-sensitive manner. In luminal breast cancer models such as MCF-7, sFRP4 enhances apoptotic activity and suppresses survival and migratory potential, consistent with inhibition of canonical Wnt signaling [81]. In the more aggressive triple-negative breast cancer context, however, the antitumor effects of sFRP4 appear to depend in part on cellular source. sFRP4 secreted by cancer-associated fibroblasts has been reported to inhibit Wnt signaling, suppress epithelial–mesenchymal transition, and reduce breast cancer cell migration, indicating that sFRP4 can also exert tumor-restraining effects through microenvironmental regulation of the tumor cell state [1].

#### 5.1.5. Urinary System Tumors

Within the urinary system, prostate cancer provides one of the most informative examples of how the biological role of sFRP4 can diverge from its apparent clinical associations. Functional studies generally support a tumor-suppressive role for sFRP4 in this disease, whereas clinical profiling studies have also linked higher sFRP4 expression to aggressive tumor features, indicating that interpretation of sFRP4 in prostate cancer depends strongly on molecular context and tissue compartment.

Mechanistically, sFRP4 downregulation in prostate cancer has been linked to both promoter hypermethylation and histone-associated repression, with DNA methylation appearing to be the dominant epigenetic mechanism. Treatment with the demethylating agent 5-aza-dC markedly restored sFRP4 mRNA expression in hypermethylated prostate cancer cell lines and cancer stem-like cells (CSCs) [38]. In addition, sFRP4 has been shown to inhibit proliferation of androgen-independent prostate cancer cells, and higher membrane-associated sFRP4 protein expression has been significantly associated with more favorable prognosis in patients with androgen-dependent prostate cancer [83]. Taken together, these findings are most consistent with a tumor-restraining function at the biological level.

A more complex picture emerges from clinical association studies. Increased sFRP4 expression has been observed in prostate cancer tissues, and subsequent studies have likewise associated elevated sFRP4 expression with aggressive disease [84,85,86]. Higher sFRP4 levels have been linked to high-grade disease, postoperative recurrence, and increased invasiveness [87]. Importantly, however, these observations do not necessarily indicate that sFRP4 exerts tumor-promoting activity. Rather, they may reflect context-dependent expression patterns associated with tumor progression, epigenetic dysregulation, or changes in tissue composition.

This interpretation is supported by spatial transcriptomic and multiomic analyses showing that sFRP4 mRNA is predominantly localized to stromal/interstitial regions of prostate cancer, with the highest expression detected in interstitial high-grade (GG3-5) cancer tissues, followed by lymphocytes, high-grade cancer cells, and interstitial low-grade cancer. In contrast, sFRP4 expression remains relatively low in normal prostate tissues [30]. Notably, despite relatively high mRNA abundance in cancer tissues, detectable sFRP4 protein levels may remain low. This transcript–protein discrepancy should be interpreted cautiously, because transcript abundance does not necessarily reflect functional protein activity. Several non-mutually exclusive factors may contribute to this inconsistency. First, increased stromal or interstitial sFRP4 mRNA expression may not directly translate into abundant detectable protein because of reduced translational efficiency or post-transcriptional regulation. Second, as a secreted protein, sFRP4 may be released into the extracellular space, diluted within the stromal matrix, or undergo extracellular degradation, thereby reducing its detection in tissue-based protein assays. Third, differences in cellular source may also influence interpretation, as stromal and tumor cells may differ in sFRP4 transcription, secretion, and local protein handling. Therefore, elevated sFRP4 mRNA, particularly in spatially heterogeneous tumor tissues, should not be directly equated with increased functional sFRP4 protein activity [30]. In this setting, elevated sFRP4 expression may therefore function more plausibly as a biomarker of an aggressive stromal or microenvironment-associated state than as direct evidence of tumor-promoting activity by sFRP4 itself.

Bladder cancer, by contrast, appears to fit a more conventional epigenetic silencing model. Clinical studies have shown that sFRP4 methylation levels are markedly higher in bladder tumor tissues than in normal bladder mucosa, and this methylation is negatively correlated with sFRP4 mRNA expression [89]. These findings support promoter hypermethylation as a major mechanism of sFRP4 loss in bladder cancer and are consistent with a predominantly tumor-suppressive interpretation in this setting.

#### 5.1.6. Nervous System Tumors

In nervous system tumors, the available evidence predominantly supports a tumor-suppressive role for sFRP4. Across glioma-related models, this effect appears to be mediated mainly through promotion of apoptotic signaling, suppression of proliferative and stem-like programs, and epigenetic regulation of sFRP4 expression.

In gliomas, sFRP4 has been reported to promote p53 activation and engage apoptosis-associated pathways. In one proposed mechanism, sFRP4 interacts with the homologous box gene Cphx1 to regulate the aging-related factor ETS2 and activates the p53 pathway effector miRNA885, thereby inducing tumor cell apoptosis through the Fas–p53–DNA damage axis [27]. Additional studies have shown that sFRP4 upregulates pro-apoptotic markers such as Bax and p21 while downregulating Cyclin D1 and the stem cell marker CD133, shifting the balance away from proliferation and toward apoptosis [90]. sFRP4 has also been associated with increased intracellular reactive oxygen species (ROS) levels, further supporting a pro-apoptotic effect [91]. Consistent with these observations, sFRP4 has been linked to enhanced sensitivity of cancer stem-like cells (CSCs) to chemotherapy, suggesting therapeutic relevance in combination treatment settings [90].

This tumor-restraining pattern extends to glioblastoma (GBM), where epigenetic silencing of sFRP4 has also been documented. Experimental studies showed that treatment with the demethylating agent 5-azacytidine restored sFRP4 expression and increased sFRP4 protein levels, supporting promoter hypermethylation as a mechanism underlying loss of sFRP4 function in GBM [92]. Beyond this epigenetic mode of suppression, sFRP4 has also been proposed to exert a less conventional nuclear role in GBM by promoting DNA damage-associated cell death, further reinforcing its antitumor activity in this setting [27].

Pituitary adenomas likewise fit a predominantly suppressive model. In aggressive pituitary adenomas, promoter methylation of sFRP4 is associated with reduced expression, and lower sFRP4 levels correlate with greater tumor aggressiveness [94]. In growth hormone-secreting pituitary adenomas, both sFRP4 mRNA and protein levels were lower in invasive than in non-invasive tumors. Tissue microarray analysis further showed that weak sFRP4 expression was more frequent in the invasive group, and low sFRP4 expression was significantly associated with invasiveness and served as an independent risk factor for recurrence or progression [95]. Increased promoter methylation in the low-sFRP4-expression group supports an epigenetic basis for this reduction. Clinically, these findings suggest that reduced sFRP4 expression may have value as an indicator of invasive behavior and adverse outcome in this tumor subtype [95].

Taken together, nervous system tumors currently provide a relatively consistent picture in which sFRP4 acts mainly as a tumor-restraining factor. The most prominent context-dependent variables in this group appear to involve the mode of pathway engagement—apoptotic, epigenetic, or stemness-related—rather than a true reversal from suppressive to tumor-promoting behavior.

#### 5.1.7. Additional Solid Tumor Contexts

Additional solid tumor contexts provide further, although more limited, support for a predominantly tumor-suppressive role of sFRP4. In cutaneous squamous cell carcinoma, the methylation frequency of the sFRP4 promoter was significantly higher than that in adjacent tissues and normal skin samples, consistent with an epigenetic silencing pattern [96]. However, the current evidence in this setting remains limited by small sample size and by a narrow analytical focus on sFRP family genes, and broader validation in larger case–control studies will be required [96].

A related functional extension of this model comes from cancer stem-like cells (CSCs), in which sFRP4-associated epigenetic regulation has been implicated across multiple epithelial tumor models, including breast, prostate, and ovarian cancer cell lines [30]. In these settings, aberrant promoter hypermethylation-mediated silencing of sFRP family genes appears to support CSC maintenance, self-renewal, drug resistance, and resistance to apoptosis. Restoration of sFRP4 expression through modulation of DNA methyltransferase- and histone-associated repression has been linked to suppression of aberrant Wnt signaling and attenuation of CSC-associated phenotypes [30]. These observations suggest that the significance of sFRP4 loss may extend beyond bulk tumor growth to stemness-associated cellular states that contribute to recurrence and treatment failure.

Together, these additional settings broaden the interpretive framework for sFRP4 in solid tumors. Although they do not yet provide the same level of mechanistic detail as the better-characterized tumor types discussed above, they suggest that sFRP4 may influence not only tumor progression itself, but also the persistence of therapy-resistant cellular subpopulations.

### 5.2. Hematologic Malignancies

In hematologic malignancies, the available evidence predominantly supports a model in which sFRP4 is epigenetically inactivated through promoter hypermethylation, resulting in loss of its Wnt-antagonistic and tumor-suppressive functions. This pattern has been reported across several leukemia subtypes, including acute promyelocytic leukemia (APL), acute myeloid leukemia (AML), acute lymphoblastic leukemia (ALL), chronic lymphocytic leukemia (CLL), and Philadelphia chromosome-positive ALL (Ph^+^ ALL) [97,98,99,100].

Within this framework, sFRP4 hypermethylation appears to have both biological and clinical relevance. In CLL, aberrant methylation has been linked to dysregulation of cancer-related pathways, including Wnt signaling, and is closely associated with disease pathogenesis [101]. In acute leukemia, sFRP4 hypermethylation has been detected in a subset of cases, with reported frequencies of 25.0% in ALL and 6.8% in AML, suggesting possible biomarker relevance, particularly in ALL [98].In APL, sFRP4 hypermethylation has also been proposed as a potential therapeutic target [97]. Moreover, in Ph^+^ ALL, sFRP4 methylation has been associated with markedly reduced survival and has been identified as an independent prognostic factor [100].

Importantly, these observations should not be interpreted as evidence of a tumor-promoting role for sFRP4 itself. Rather, promoter hypermethylation leads to transcriptional silencing of sFRP4, thereby abolishing its normal inhibitory influence on Wnt signaling and favoring persistent pathway activation. In this setting, disease progression is more plausibly linked to loss of sFRP4 function than to any intrinsic oncogenic activity of the protein. Overall, promoter hypermethylation appears to represent the predominant mode of sFRP4 dysregulation in hematologic malignancies.

## 6. Clinical Application of sFRP4

sFRP4 has diverse clinical relevance across hematologic malignancies and solid tumors, with potential applications in diagnosis, prognosis, risk stratification, and therapy. Importantly, these clinical utilities are highly context-dependent and may rely on distinct molecular readouts, including promoter methylation, tissue expression, and circulating protein levels (Table 2).

### 6.1. Diagnostic Value of sFRP4

Current evidence suggests that sFRP4 has potential value as a biomarker candidate in selected hematologic malignancies and solid tumors, although its performance varies substantially according to tumor type and assay modality.

In hematologic malignancies, the most prominent diagnostic signal involves epigenetic alteration as preclinical evidence. In the NB4 acute promyelocytic leukemia (APL) cell line, mechanistic analyses demonstrated marked hypermethylation of the sFRP4 promoter region. Bisulfite sequencing PCR and cloning sequencing further showed that the methylation level of sFRP4 reached 85% in NB4 cells, compared with 2.5% in healthy controls *p* < 0.01 [97]. This pronounced methylation difference suggests that sFRP4 hypermethylation may have preclinical biomarker relevance in APL, while also supporting the translational hypothesis that sFRP4 silencing is associated with early leukemogenic events [97].

Regarding circulating biofluids, serum detection highlights that the biomarker relevance of sFRP4 represents a statistical clinical association that depends heavily on the specific biological sample and tumor type being assessed. In hepatocellular carcinoma (HCC), serum sFRP4 levels were reported to be significantly higher than those in non-cancer controls, indicating its preliminary exploration as a circulating biomarker candidate [58]. By contrast, opposite serological trends have been reported in endometrial and ovarian cancers, where reduced serum sFRP4 levels may help distinguish patients with these malignancies from healthy individuals [52]. These findings underscore the tissue- and cancer-specific heterogeneity of sFRP4 as a context-dependent clinical association rather than a validated diagnostic indicator.

Beyond single-marker applications, sFRP4 has also been incorporated into multimodal in silico frameworks. Machine learning-based feature selection using random forest, LASSO, and SVM-RFE identified sFRP4 as a core predictive gene for HCC, and it was subsequently incorporated into an artificial neural network model [59]. This finding suggests that the predictive performance of sFRP4 may be strengthened when integrated into multigene prediction systems rather than interpreted in isolation. In addition, a combined molecular signature including sFRP4, ECM2, METTL7B, and MNS1 showed good discriminatory performance for heart failure in patients with lung cancer [103]. Although this latter setting extends beyond primary tumor diagnosis, it suggests that sFRP4 may also participate in biomarker panels for tumor-associated systemic complications, representing a therapeutic and diagnostic strategy that is not yet clinically validated.

Overall, the biomarker relevance of sFRP4 lies not in a universal readout, but in its context-specific application across distinct molecular and clinical settings. At present, its most plausible translational hypotheses include exploratory epigenetic testing in hematologic malignancies, serum-based adjunctive evaluation in selected solid tumors, and incorporation into composite predictive models.

### 6.2. Prognostic Value and Risk Stratification of sFRP4

The prognostic relevance of sFRP4 is strongly context-dependent, presenting as a statistical clinical association that depends on the biological state it reflects rather than a universally applicable prognostic indicator. In tumors conforming to the conventional tumor-suppressive model, prognostic characterization heavily relies on sFRP4 loss driven by transcriptional and post-transcriptional dysregulation, which serves as a potential biomarker candidate. At the post-transcriptional level, preclinical evidence in pancreatic carcinogenesis indicates that malignant transformation is accompanied by aberrant upregulation of miR-135b-5p, establishing a miRNA-mediated negative regulatory axis that drives sFRP4 downregulation and correlates with adverse pathological features and reduced survival [43]. Consistently, at the transcriptional and mRNA levels, reduced sFRP4 mRNA expression closely parallels higher histological grade, local recurrence, or distant metastasis in endometrioid adenocarcinoma [51], while the loss of membranous sFRP4 protein extends as a prognostic correlation to broader gynecological malignancies like endometrial and ovarian cancers [52].

Conversely, a paradoxical clinical association is observed in other contexts where elevated sFRP4 mRNA and protein enrichment reflect a tumor-promoting microenvironmental state. In gastric cancer, retrospective clinical data showed that a high-sFRP4-expression state statistically correlates with a substantially lower 5-year survival rate (39.81% versus 60.02% in the low-expression cohort) [66]. Mechanistically, this high sFRP4 level is positively associated with PD-L1 expression, promoter demethylation-associated invasion [6], and CD8^+^ T-cell infiltration, suggesting it marks a distinct immune-related tumor state rather than a simple gain of suppressive activity [66]. A similar microenvironmental clinical association occurs in pancreatic ductal adenocarcinoma (PDAC), where higher sFRP4 expression links to FOXP3^+^ regulatory T-cell infiltration, impaired effector T-cell recruitment, and shortened survival [73].

Furthermore, sFRP4 has been integrated into multimodal in silico prognostic models for risk stratification. These include a combined framework with immune markers (e.g., GZMB) in gastric cancer [67], an extracellular matrix-related five-gene signature in PDAC [76], an EMT-related six-gene signature for metastatic prediction in triple-negative breast cancer [82] and the EMTGPI scoring system in prostate cancer [88]. Collectively, these findings indicate that the translational value of sFRP4 in prognosis represents a translational hypothesis that is not yet clinically validated, emphasizing its utility not as an isolated prognostic parameter, but as a component within broader, context-specific molecular frameworks.

### 6.3. Therapeutic Potential and Translational Challenges of sFRP4

The therapeutic relevance of sFRP4 is highly context-dependent and cannot be reduced to a uniform strategy of either activation or inhibition. In tumors where sFRP4 is epigenetically silenced or functionally repressed, restoration of its activity may help re-establish tumor-suppressive signaling. By contrast, in selected settings associated with chemoresistance, elevated sFRP4 appears to accompany tumor-promoting programs and may instead represent a target for inhibition. These contrasting patterns indicate that the translational value of sFRP4 lies in mechanism-guided intervention rather than a single therapeutic paradigm.

In tumors characterized by promoter hypermethylation-mediated or post-transcriptional silencing of sFRP4, restoration strategies appear particularly relevant. Similar silencing of sFRP4 has been reported in uterine sarcoma [57], oral squamous cell carcinoma (OSCC) [78] and endometrial cancer [104], suggesting that epigenetic or transcriptional repression of sFRP4 may define a therapeutically reversible state in multiple malignancies. In addition to DNA methylation, upstream non-coding RNA regulation may also be targetable. In esophageal squamous cell carcinoma (ESCC), miR-942 directly targets and inhibits sFRP4, indicating that blockade of specific upstream repressors may represent a feasible strategy to restore sFRP4 activity and restrain Wnt signaling [44].

The tumor-suppressive effects of sFRP4 have also been explored through exogenous replacement and domain-based therapeutic approaches. Recombinant human sFRP4 protein (rhsFRP4), produced using a prokaryotic expression system, inhibited the proliferation of HeLa cervical cancer cells and A549 lung cancer cells in a dose-dependent manner, with a maximum inhibitory rate of approximately 40% in vitro [105]. Combined treatment with rhsFRP4 and conventional chemotherapeutic agents further enhanced antitumor efficacy in cell culture lines, supporting the conceptual feasibility of hypothetical combination designs rather than an actionable clinical strategy [105]. Functional analyses of distinct sFRP4 domains further suggest that specific regions of the protein may be therapeutically exploitable. Peptides derived from the cysteine-rich domain (CRD) and netrin-like domain (NLD), including SC-301 and SC-401, substantially reduced the migratory capacity of ovarian cancer stem cells [54]. In addition, SC-401 reversed epithelial–mesenchymal transition, as evidenced by downregulation of mesenchymal markers and upregulation of E-cadherin [54]. Recombinant sFRP4 also induced phosphorylation and inactivation of β-catenin and suppressed downstream oncogenic targets, including cyclin D1, c-myc, and survivin, ultimately leading to G2/M phase arrest and early apoptosis [54]. Beyond canonical Wnt inhibition, the NLD may exert anti-invasive effects through additional mechanisms. In glioblastoma, sFRP4-NLD inhibited MMP-2 activity and disrupted fibronectin assembly in LN229 cells, thereby attenuating extracellular matrix remodeling and invasive behavior [93].

In contrast, a different therapeutic logic may apply in tumors in which sFRP4 is linked to chemoresistance. In gastric cancer, sFRP4 has been implicated in signaling rewiring associated with tolerance to cisplatin and oxaliplatin, and higher expression has been associated with poor prognosis [68]. Similarly, the miR-181a–sFRP4 axis has been reported to support stemness maintenance and platinum resistance in ovarian cancer [55]. These observations suggest that, in selected resistant tumors, sFRP4-related signaling may need to be blocked rather than restored. Thus, depending on the molecular context, sFRP4 may represent either a restorative target or an inhibitory target.

Despite these promising findings, substantial translational challenges remain. The foremost issue is functional duality: sFRP4 may act as a tumor suppressor in some malignancies yet accompany tumor-promoting or drug-resistant states in others. This complexity makes biomarker-guided patient selection essential. Additional long-term theoretical challenges include the hypothetical choice of conceptual delivery modalities, such as epigenetic drugs, non-coding RNA-targeted interventions, recombinant proteins, or domain-derived peptides, as well as the strict requirement for robust in vivo validation in preclinical animal models long before any future clinical consideration. Future exploratory investigation of sFRP4-based mechanistic concepts will therefore require precise molecular stratification, careful matching of intervention strategy to tumor context, and rigorous preclinical evaluation of hypothetical delivery feasibility and targeting specificity.

### 6.4. Systemic Metabolic Regulation and Phosphate Homeostasis

Beyond its tumor-intrinsic functions, sFRP4 has also been implicated in tumor-associated systemic metabolic dysregulation, particularly in tumor-induced osteomalacia (TIO). As a member of the phosphatonin family, sFRP4 has attracted attention for its potential contribution to phosphate wasting and disturbed mineral metabolism in this rare paraneoplastic syndrome [102,106]. TIO is typically caused by mesenchymal tumors that secrete phosphaturic factors, and available evidence suggests that sFRP4 may participate in this endocrine disturbance together with other circulating mediators.

Mechanistically, elevated sFRP4 has been associated with two major abnormalities relevant to TIO pathophysiology. First, it may impair phosphate reabsorption in the proximal renal tubules, thereby contributing to renal phosphate wasting. Second, it has been reported to suppress the biosynthesis of active vitamin D 1α,25(OH)_2_D through inhibition of 25-hydroxyvitamin D-1α-hydroxylase activity [102,107]. Together, these effects may contribute to refractory hypophosphatemia and defective bone mineralization, which characterize the biochemical and skeletal manifestations of TIO.

An important translational paradox, however, complicates the interpretation of sFRP4 in this setting. Although elevated circulating sFRP4 levels in patients with TIO have been associated with the severity of phosphate metabolic disturbance, sFRP4 knockout mice do not display the expected abnormalities in phosphate homeostasis and instead show a mild or absent phenotype [108]. This discrepancy suggests that the phosphaturic activity attributed to sFRP4 may not be fully autonomous, but may depend on tumor-specific signals, permissive microenvironmental conditions, or cooperation with additional endocrine cofactors.

Accordingly, current evidence supports a network-based rather than single-factor model of TIO pathophysiology. sFRP4 is unlikely to act independently; instead, it may function in concert with fibroblast growth factor 23 (FGF23) and extracellular matrix phosphoglycoprotein-related factors within a broader phosphaturic regulatory network [109]. In this context, sFRP4 may have value not only as a potential adjunctive serum biomarker of TIO, but also as a mechanistic link between tumor-derived signals and remote systemic metabolic reprogramming. More broadly, the study of sFRP4 in TIO provides a useful framework for understanding how tumors can influence host mineral metabolism through endocrine and microenvironment-dependent pathways.

## 7. Discussion and Prospects

The available evidence indicates that sFRP4 is not a static tumor suppressor, but a highly context-dependent regulator whose biological and clinical significance varies according to tumor type, molecular subtype, epigenetic state, and microenvironmental context. This functional plasticity represents both the greatest scientific interest and the main translational challenge of sFRP4 research. In some malignancies, loss of sFRP4 expression is associated with Wnt pathway activation, aggressive tumor behavior, and poor outcome, consistent with a classical tumor-suppressive model. In other settings, however, elevated sFRP4 expression correlates with immune evasion, chemoresistance, or unfavorable prognosis. Thus, the central challenge is not simply whether sFRP4 is “beneficial” or “harmful,” but under which biological conditions it assumes either role.

This duality likely arises from the interaction between the intrinsic molecular properties of sFRP4 and the extrinsic regulatory pressures imposed by the tumor microenvironment. Beyond its canonical role as a Wnt antagonist, sFRP4 may participate in immune remodeling, extracellular matrix reorganization, and epithelial–mesenchymal transition in a tumor-specific manner. In tumor-cell-intrinsic tumor-suppressive contexts, promoter hypermethylation, transcriptional repression, or upstream non-coding RNA regulation may reduce sFRP4 expression, thereby weakening its restraint on canonical Wnt/β-catenin signaling. β-catenin accumulation, activation of Wnt-responsive genes, increased proliferation, enhanced invasion, and reduced treatment sensitivity are more consistent with tumor-promoting consequences driven by loss of sFRP4 function. By contrast, in immune-remodeled or therapy-resistant contexts, sFRP4 expression is frequently increased and correlated with tumor promotion and unfavorable clinical outcomes. However, this tumor-supportive correlation and elevated expression should not be simply interpreted as direct evidence that sFRP4 itself acts as a cell-autonomous oncogenic driver. More accurately, elevated sFRP4 in these settings accompanies, reflects, or participates in a permissive tumor microenvironmental state that favors tumor progression, which is functionally characterized by chronic inflammatory or immune-suppressive signaling, regulatory T-cell infiltration, chemoresistance, and adverse prognosis. Gastric cancer illustrates this shift particularly well: high sFRP4 expression is associated with PD-L1/PD-1-related immunosuppressive features and poor survival, suggesting that its immunomodulatory effects may outweigh its classical Wnt-inhibitory activity in chronic inflammatory and fibroproliferative settings [66]. A similar context-dependent reversal is seen in prostate cancer, where the prognostic meaning of sFRP4 differs according to molecular subtype, particularly ERG status [88]. In pancreatic cancer, progressive upregulation of sFRP4 during tumor evolution and its association with FOXP3^+^ Treg infiltration further support the view that sFRP4 may be co-opted by advanced tumors to sustain an immunosuppressive microenvironment [73]. Together, these findings suggest that the biological effect of sFRP4 is determined less by its isolated expression level than by the signaling networks and cellular ecosystems in which it operates.

This context dependence has direct implications for clinical translation. Strategies aimed at restoring sFRP4 expression may be rational in tumors characterized by promoter hypermethylation or transcriptional silencing, whereas inhibition of sFRP4-related signaling may be more appropriate in tumors in which sFRP4 is linked to immune escape or chemoresistance. Accordingly, future therapeutic development should avoid a uniform strategy of indiscriminate sFRP4 activation or suppression. Instead, intervention must be guided by tumor-specific molecular stratification, including epigenetic state, immune contexture, and subtype-specific signaling features. In this regard, sFRP4 is best viewed not as a universal therapeutic target, but as a context-sensitive node within broader regulatory networks.

Looking ahead, several long-term research priorities remain at an early conceptual stage for exploring the potential translational insights of sFRP4. First, robust laboratory frameworks are needed to clarify the specific biological contexts under which sFRP4 functions as a suppressive versus tumor-promoting factor, including standardized experimental assessment of promoter methylation, tissue expression, and circulating levels. Second, more physiologically relevant models, such as patient-derived organoids, co-culture systems, and humanized tumor microenvironment platforms, will be essential to dissect the dynamic, context-specific effects of sFRP4 in experimental settings that better mimic human disease. Third, future exploratory development should focus on early-stage mechanistic optimization, including recombinant protein characterization, domain-based peptide modeling, epigenetic reactivation studies, and hypothetical delivery vectors designed to enhance tissue targeting and stability in preclinical models [2]. Finally, rigorous preclinical validation will be critical to determine whether sFRP4-targeted conceptual strategies can provide reproducible evidence in specific laboratory cohorts before any future clinical consideration. With these stepwise investigations, sFRP4 may gradually evolve from a biologically intriguing Wnt antagonist into a better-characterized candidate for future precision oncology research.

## Figures and Tables

**Table 1:** Evidence summary of sFRP4 across different cancer types and contexts.

Cancer Type	Functional Role	Detection Method	Evidence Type	Limitations	References
Cervical cancer	Tumor-suppressive	Promoter methylation; DNMT1 knockdown; 5-aza-dC treatment	Clinical association (stepwise methylation increase in lesions); cell-based mechanistic studies	Methylation status not significantly associated with clinical stage; requires prospective validation	[34,48,49]
Endometrial cancer	Tumor-suppressive	PBX1/sFRP4 axis; mRNA/protein expression	Cell-based mechanistic studies (invasion, EMT suppression)	Primarily cell line data; in vivo validation limited	[50]
	Prognostic (favorable)	mRNA expression	Clinical association (favorable prognostic biomarker)	Retrospective analysis; requires independent cohort validation	[51]
Endometrial & ovarian cancers	Diagnostic (serum)	Serum sFRP4 protein	Clinical association (reduced levels in patients vs. healthy controls)	Requires assay standardization; independent validation needed	[52]
Ovarian cancer	Tumor-suppressive	Recombinant sFRP4 treatment; recombinant protein/peptide functional assays (proliferation/migration/angiogenesis/chemosensitization); pathway detection; EMT markers	Cell-based mechanistic studies	In vivo validation lacking	[53,54,55]
DES-induced uterine adenocarcinoma (mouse model)	Observational (not conclusive)	Gene expression	Animal model (correlation evidence)	Observational association; does not establish a direct tumor-promoting function; mechanistic proof lacking	[56]
Uterine sarcoma	Tumor-suppressive	Immunohistochemistry (IHC); recombinant SFRP4 protein treatment	Preclinical cell line experiments and mechanistic studies	Very small sample size	[57]
Esophageal adenocarcinoma/Barrett’s esophagus	Tumor-suppressive	Promoter methylation	Clinical association (early event in Barrett’s-EAC sequence)	Predominantly methylation frequency; limited functional validation	[39]
Hepatocellular carcinoma	Tumor-suppressive	sFRP4 overexpression; GSK-3β/β-catenin modulation	Cell-based mechanistic studies	In vivo validation limited	[16]
	Diagnostic (serum)	Serum sFRP4 protein	Clinical association (higher levels in HCC patients vs. controls)	Requires assay standardization; independent validation needed	[58]
	Not functionally evaluated	Multi-gene signature (bioinformatics)	Pure bioinformatics analysis; no functional experiments	No cell/animal experimental validation	[59]
Gastric cancer	Tumor-suppressive (methylation context)	Promoter methylation	Clinical association	Methylation-mediated silencing contributes to Wnt activation	[60,61,62,63,64]
	Tumor-promoting (demethylation/PKA context)	Promoter demethylation; PKA phosphorylation; PD-L1 expression; CD8^+^ T-cell infiltration	Clinical association; cell-based mechanistic studies	Complex regulatory mechanisms; requires further mechanistic dissection	[6,29,65,66]
	Prognostic (adverse)	Protein expression; PD-L1; 5-year survival	Clinical association	Retrospective analysis; requires prospective validation	[66]
	Tumor-promoting (clinical association)	Immunohistochemistry; survival correlation; clinicopathological feature association	Mixed evidence from mechanistic research and clinical associations	SFRP4 function not validated in cell or animal models	[64]
	Not functionally evaluated	Multi-gene prognostic signature	Clinical association; prognostic modeling	No functional validation	[67]
	Tumor-promoting	Clinicopathological feature association; recombinant protein functional assays (migration/EMT); pathway detection	Mixed evidence from mechanistic research and clinical associations	Further mechanistic dissection needed	[68]
Colorectal cancer	Tumor-suppressive (general)	Promoter methylation; mRNA expression	Clinical association	Correlation not causation	[69,70]
	Tumor-promoting (mesenchymal subtype)	mRNA expression; EMT gene co-expression	Clinical association (worse survival)	Subtype-specific finding; requires mechanistic validation	[71]
	Tumor-promoting (bioinformatics)	Transcriptomic analysis; EMT marker co-expression analysis; survival correlation	Pure bioinformatics analysis; no functional validation	No cell/animal/tissue experiments	[71]
Pancreatic ductal adenocarcinoma	Tumor-suppressive (epithelial compartment)	Promoter methylation; miR-135b-5p targeting; mRNA expression	Clinical association; cell-based mechanistic studies	Epithelial vs. stromal compartments require separate interpretation	[43,72]
	Tumor-promoting (stromal compartment)	Tissue mRNA; stromal/interstitial localization; Treg infiltration; ASC-derived sFRP4	Clinical association; animal models; spatial transcriptomics; cell-based studies	Tumor-supportive correlation should not be equated with direct oncogenic activity of sFRP4 itself	[73,74,75]
	Prognostic (adverse)	mRNA expression; immune markers (FOXP3^+^ Treg)	Clinical association	Retrospective analysis; requires prospective validation	[73]
	Not functionally evaluated	Multi-gene prognostic signature	Clinical association; prognostic modeling	No functional validation	[76]
Oral squamous cell carcinoma	Tumor-suppressive	Promoter methylation	Clinical association	Small sample size; limited functional validation	[77,78]
Malignant mesothelioma	Tumor-suppressive	Promoter methylation; Akt/GSK-3β pathway; tissue/cell line mRNA expression	Clinical association; cell-based mechanistic studies	In vivo validation limited; small sample size	[26,79,80]
Breast cancer	Tumor-suppressive (compartment-dependent)	CAF-secreted sFRP4; apoptosis/EMT markers	Cell-based studies	Different subtypes and cellular sources may yield different effects	[1,81]
	Tumor-suppressive	Single-cell RNA sequencing; public database analysis	Single-cell sequencing; spatial transcriptomics; prognostic survival analysis	Small sample size (only 7 patients); SFRP4 as a secreted protein is difficult to study directly	[1]
	Prognostic (metastatic, TNBC)	EMT-related six-gene signature	Clinical association; prognostic modeling	Bioinformatics analysis; requires experimental validation	[82]
Prostate cancer	Tumor-suppressive (functional studies)	Promoter methylation; histone-associated repression; membrane protein loss	Clinical association; cell-based studies	Functional role vs. clinical association appear contradictory	[38,83]
	Tumor-promoting (clinical association)	Stromal/interstitial mRNA (spatial transcriptomics)	Clinical association (aggressive disease, recurrence)	Elevated mRNA should not be equated with increased functional protein activity; transcript–protein discrepancy	[30,84,85,86,87]
	Prognostic (ERG status-dependent)	mRNA; EMTGPI scoring	Clinical association (prognostic meaning differs by ERG fusion status)	Molecular subtype-specific; requires validation	[88]
Bladder cancer	Tumor-suppressive	Promoter methylation	Clinical association	Preliminary evidence; requires further validation	[89]
Glioma/Glioblastoma	Tumor-suppressive	Promoter methylation; Fas-p53 axis; ROS; apoptosis	Cell-based mechanistic studies	In vivo validation limited	[27,90,91,92]
	Tumor-suppressive	Overexpression functional assays (stemness/invasion/migration/adhesion/ECM degradation); pathway detection	Cell-based mechanistic studies	No in vivo tumor model validation (in vitro only)	[93]
Pituitary adenoma	Tumor-suppressive	Promoter methylation; mRNA/protein expression	Clinical association (low sFRP4 associated with invasiveness and recurrence)	Limited sample size; requires independent validation	[94,95]
Cutaneous squamous cell carcinoma	Tumor-suppressive	Promoter methylation; clinicopathological feature association; diagnostic marker evaluation	Clinical methylation analysis; methylation-expression correlation and partial functional assessment	Only Han Chinese population in Xinjiang, China; limited ethnic representation	[96]
Hematologic malignancies	Tumor-suppressive (APL/AML/ALL)	Promoter methylation	Clinical association	Predominantly methylation frequency; functional validation limited	[97,98,99,100]
	Tumor-suppressive (multi-cell lines)	Promoter methylation	Multi-cell line/multi-gene methylation profiling	No primary patient specimen validation	[99]
	Tumor-suppressive (CLL)	Promoter methylation (clinical specimens); multi-gene methylation	Clinical methylation analysis; methylation-expression correlation	Small sample size	[101]
	Tumor-suppressive (Ph^+^ ALL)	Promoter methylation; clinicopathological feature association; survival correlation	Clinical methylation analysis; methylation-expression correlation	Further validation in independent cohorts needed	[100]

**Table 2:** Summary of cancer biomarkers by type and clinical relevance.

Biomarker Type	Detection Dimension	Cancer Type	Clinical Significance	Reference
Diagnostic Biomarker	Methylation status	APL	Potential epigenetic biomarker for early screening and differentiation of APL from healthy individuals.	[97]
		AML & ALL	Facilitates early detection and serves as an indicator for disease onset risk.	[98]
		EAC & BE	Distinguishes precancerous lesions from malignant tissues; a core indicator for early diagnosis.	[39]
		CC	Complements Pap smear limitations; combined with sFRP family members to enhance screening sensitivity.	[48]
		OSCC	Significantly associated with OSCC development; serves as an auxiliary diagnostic tool.	[77]
		MM	A characteristic epigenetic aberration utilized for the diagnosis of MM.	[79]
		BC	Discriminates malignant from benign bladder tissues for auxiliary diagnosis.	[89]
		GBM	Diagnostic reference for GBM, indicating underlying epigenetic abnormalities.	[92]
	Protein expression (Tissue & Serum)	HCC	Enhances diagnostic accuracy when combined with AFP; indicates disease progression.	[58]
		EC & OC	Sensitively discriminates healthy individuals from patients to aid gynecological cancer diagnosis.	[52]
Prognostic Biomarker	Protein expression & Methylation status	GC	Mediates immune evasion; predicts disease progression and poor prognosis in GC.	[66]
		CRC	Reflects EMT-driven malignant progression; indicates high metastatic risk and poor prognosis.	[71]
		PDAC	An adverse prognostic biomarker associated with the mediation of immune evasion status.	[73]
		CRC	Potential poor prognostic factor indicating a high risk of recurrence.	[71]
		EC	Favorable prognostic biomarker	[51]
Therapeutic Biomarker	Dynamic changes in methylation & Protein expression	PCa	Restores epigenetic silencing to drive anti-Wnt precision therapy.	[38]
		Glioma	Evaluates chemo-sensitivity and predicts therapeutic response.	[90]
	Protein expression & PD-L1 levels	GC	Identifies immune targets and guides combination therapy regimens.	[66]
	Protein expression & Platinum-sensitivity	HGSOC	Identifies platinum-resistance targets to guide resistance-reversal strategies.	[55]
Other Specific Biomarker	Protein expression (Serum)	TIO	Potential serological indicator for TIO	[102]

APL: Acute Promyelocytic Leukemia; AML: Acute Myeloid Leukemia; ALL: Acute Lymphoblastic Leukemia; EAC: Esophageal Adenocarcinoma; BE: Barrett’s Esophagus; CC: Cervical Cancer; sFRP: Secreted Frizzled-Related Protein; OSCC: Oral Squamous Cell Carcinoma; MM: Multiple Myeloma; BC: Bladder Cancer; GBM: Glioblastoma Multiforme; HCC: Hepatocellular Carcinoma; AFP: Alpha-fetoprotein; EC: Endometrial Cancer; OC: Ovarian Cancer; GC: Gastric Cancer; CRC: Colorectal Cancer; EMT: Epithelial–Mesenchymal Transition; PDAC: Pancreatic Ductal Adenocarcinoma; PCa: Prostate Cancer; Wnt: Wingless-related integration site; PD-L1: Programmed Death-Ligand 1; HGSOC: High-Grade Serous Ovarian Cancer; TIO: Tumor-induced Osteomalacia.

## Data Availability

No new data were created or analyzed in this study. Data sharing is not applicable to this article.

## Abbreviations

The following abbreviations are used in this manuscript:

sFRP4	secreted frizzled-related protein 4
sFRPs	secreted frizzled-related proteins sFRPs
TME	tumor microenvironment
CRD	cysteine-rich domain
FZD	Frizzled
GSK-3β	glycogen synthase kinase-3β
JNK	c-Jun N-terminal kinase
NLD	netrin-like domain
TFs	transcription factors
DNMT	DNA methyltransferase
CSCs	cancer stem cells
EAC	esophageal adenocarcinoma
PDAC	pancreatic ductal adenocarcinoma
3′-UTR	3′ untranslated region
ESCC	esophageal squamous cell carcinoma
SCC	squamous cell carcinoma
5-aza-dC	5-aza-2′-deoxycytidine
EMT	epithelial–mesenchymal transition
rsFRP4	recombinant sFRP4 protein
PanIN	pancreatic intraepithelial neoplasia
ASCs	adipose stromal cell
PSCs	pancreatic stellate cells
HCC	hepatocellular carcinoma
OSCC	oral squamous cell carcinoma
TCF/LEF	T-cell factor/lymphoid enhancer factor
MM	malignant mesothelioma
GBM	glioblastoma
APL	acute promyelocytic leukemia
AML	acute myeloid leukemia
ALL	acute lymphoblastic leukemia
CLL	chronic lymphocytic leukemia
AFP	alpha-fetoprotein
NACT	neoadjuvant chemotherapy
rhsFRP4	recombinant human sFRP4 protein
TIO	tumor-induced osteomalacia
FGF23	fibroblast growth factor 23
CS–DS	chitosan–glucan sulfate
LRP5/6	low-density lipoprotein receptor-related protein 5/6
PLC	phospholipase C
IP_3_	inositol 1,4,5-trisphosphate
PKC	protein kinase C
NFAT	nuclear factor of activated T-cells
PD-L1	Programmed Death-Ligand 1
PD-1	Programmed Cell Death Protein 1
DKK3	Dickkopf WNT signaling pathway inhibitor 3
METTL7B	Methyltransferase like 7B
MNS1	Meiosis Specific Nuclear Structural 1
MMP	matrix metalloproteinase
BE	Barrett’s Esophagus
CC	Cervical Cancer
BC	Bladder Cancer
EC	Endometrial Cancer
OC	Ovarian Cancer
GC	Gastric Cancer
CRC	Colorectal Cancer
PCa	Prostate Cancer
HGSOC	High-Grade Serous Ovarian Cancer
